# Regularization Meets Enhanced Multi-Stage Fusion Features: Making CNN More Robust against White-Box Adversarial Attacks

**DOI:** 10.3390/s22145431

**Published:** 2022-07-20

**Authors:** Jiahuan Zhang, Keisuke Maeda, Takahiro Ogawa, Miki Haseyama

**Affiliations:** 1 Graduate School of Information Science and Technology, Hokkaido University, N-14, W-9, Kita-ku, Sapporo 060-0814, Hokkaido, Japan; zhang@lmd.ist.hokudai.ac.jp; 2 Faculty of Information Science and Technology, Hokkaido University, N-14, W-9, Kita-ku, Sapporo 060-0814, Hokkaido, Japan; maeda@lmd.ist.hokudai.ac.jp (K.M.); ogawa@lmd.ist.hokudai.ac.jp (T.O.)

**Keywords:** adversarial defense, adversarial attack, feature enhancement, feature regularization

## Abstract

Regularization has become an important method in adversarial defense. However, the existing regularization-based defense methods do not discuss which features in convolutional neural networks (CNN) are more suitable for regularization. Thus, in this paper, we propose a multi-stage feature fusion network with a feature regularization operation, which is called Enhanced Multi-Stage Feature Fusion Network (EMSF^2^Net). EMSF^2^Net mainly combines three parts: multi-stage feature enhancement (MSFE), multi-stage feature fusion (MSF^2^), and regularization. Specifically, MSFE aims to obtain enhanced and expressive features in each stage by multiplying the features of each channel; MSF^2^ aims to fuse the enhanced features of different stages to further enrich the information of the feature, and the regularization part can regularize the fused and original features during the training process. EMSF^2^Net has proved that if the regularization term of the enhanced multi-stage feature is added, the adversarial robustness of CNN will be significantly improved. The experimental results on extensive white-box attacks on the CIFAR-10 dataset illustrate the robustness and effectiveness of the proposed method.

## 1. Introduction

Since deep learning technologies represented by a convolutional neural network (CNN) were proposed, the field of computer vision (e.g., image classification, object detection, and image retrieval) has developed rapidly. However, as the application range of CNNs broadens, its safety and robustness have significantly attracted the attention of academia and industry. CNNs highly depend on data, i.e., CNNs are fragile to some extent since the complexity of the data will directly affect the classification accuracy of the CNN. In 2014, Szegedy et al. [1] pointed out that if someone adds a perturbation to the original image that is sufficiently small that the human eyes cannot distinguish it, the accuracy of CNN will decrease significantly. An image added by these perturbations is called an adversarial example.

The concept of adversarial examples has attracted significant attention from related researchers since the existing CNN architectures may have huge loopholes. Furthermore, the existence of adversarial examples is a serious threat to the application of CNNs in fields of security and privacy [2]. Regarding the reasons for the existence of adversarial examples, the researchers are still in the preliminary stage of exploration, and they have discussed some possible explanations so far. Among these reasons, the idea proposed by IIyas et al. [3] is relatively novel. They considered that the adversarial examples result from sensitive features learned by the CNN. In other words, the CNN provides unrobust features.

Many excellent methods have emerged for adversarial defense, and these methods are mainly divided into four categories. The first is adversarial training [4,5,6,7,8,9]. These methods, where some subtle perturbations are added to the input data during the training process, can force CNN to adapt to these perturbations to improve the adversarial robustness. The second is to process the input data, and these methods are designed to compress [10,11], denoise [12,13,14,15], and transform [16,17,18,19] the input data to remove the adversarial noise. With the popularity of the knowledge distillation [20], some related researchers have introduced this technology into adversarial defense [21,22,23], achieving good defense effects. The latest ones are the regularization-based methods [24,25,26,27,28]. These methods help CNNs avoid overfitting and prevent the model from being too sensitive to small perturbations in the input data.

Among these methods, the regularization-based adversarial defense methods are becoming more important because of their effectiveness and low computational cost. However, there are several features in CNNs. These existing regularization-based methods do not discuss in depth what type of features are more suitable for regularization to further improve the adversarial robustness of CNNs.

In this paper, we propose a new CNN architecture called Enhanced Multi-Stage Features Fusion (EMSF^2^Net). EMSF^2^Net consists of three core operations: multi-stage features enhancement (MSFE), multi-stage features fusion (MSF^2^), and regularization. For the MSFE part inspired by SENet [29], we first perform the global average pooling (GAP) operation on the features of each stage to obtain the channel-level global features. Then, we multiply the channel-level global features with the original features to obtain the enhanced features. In the MSF^2^ part, we first flatten the enhanced multi-stage features directly into one-dimensional features. Then, we directly perform the concatenation operation on them. Although this operation is simple, it is very effective, since MSF^2^ can keep the global information on each channel learned by MSFE. Finally, we perform the regularization operation on the obtained fusion and original multi-stage features in the training process. Specifically, we use a regularization loss function as the regularization operation of EMSF^2^Net. The proposed EMSF^2^Net confirms that adding the regularization term of the enhanced multi-stage fusion feature can significantly improve the adversarial robustness of CNN. It also shows that the enhanced multi-stage fusion feature is more suitable for regularization. Furthermore, compared with existing global information-based adversarial defense approaches, we introduce the regularization technique into the fused global features and demonstrate that the regularized fused global features can further improve the adversarial robustness of CNN.

The contributions of this study are summarized as follows:

- We propose a new network, EMSF^2^Net. The enhanced multi-stage fusion feature in EMSF^2^Net can represent and keep the global information of each channel well.
- We show that regularizing the enhanced multi-stage fusion feature can significantly improve the adversarial robustness of a CNN.
- The extensive experimental results on white-box attacks with different settings show the effectiveness and robustness of the proposed approach.

## 2. The Proposed Method

**Figure 1:**
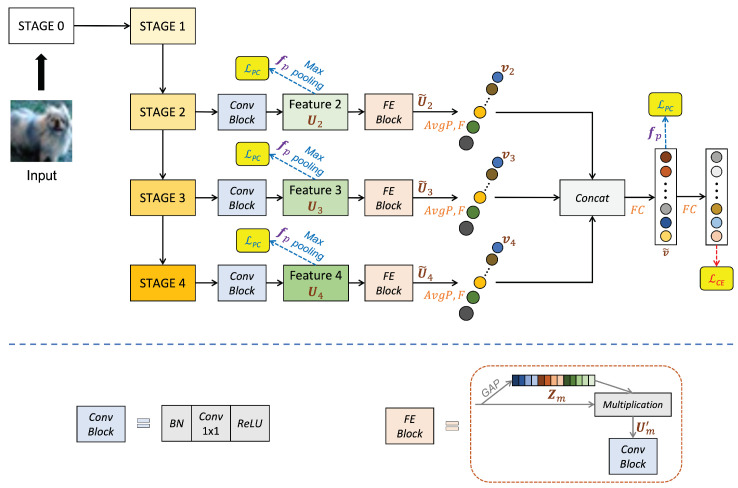
Architecture details of the proposed EMSF^2^Net. We use ResNet50 [30] as the backbone of the proposed EMSF^2^Net. The structures of STAGES 0–4 to are the same as those in baseline, where their details are presented in Table 1. In this figure, the FE Block represents the feature enhancement block; the Concat represents the concatenation operation; and the GAP in the FE Block represents the global average pooling.

Figure 1 and Table 1 show the architecture of the proposed EMSF^2^Net and the baseline, respectively. As shown in Figure 1, we use the outputs of STAGES 2–4, whose details are presented in Table 1, of the standard ResNet50 [30] as multi-stage features. The proposed EMSF^2^Net consists of three core parts: MSFE, MSF^2^, and regularization. We will explain these three parts in detail in the following subsections.

### 2.1. Multi-Stage Features Enhancement (MSFE)

In this subsection, we explain MSFE, and the details of this part are shown in the FE Block in Figure 1. Suppose the output feature after the Conv Block in STAGE *m* (*m* = 2, 3, 4) is $U_{m}=\left[u_{m}^{1},u_{m}^{2},\cdots ,u_{m}^{C_{m}}\right]\in R^{H_{m}\times W_{m}\times C_{m}}$ represented by Feature *m* (*m* = 2, 3, 4) in Figure 1. $H_{m}$, $W_{m}$, and $C_{m}$ represent the height, width, and the number of channels of Feature *m*, respectively. Furthermore, $u_{m}^{l}$ ($l=1,\cdots ,C_{m}$) represents the sub-feature of feature $U_{m}$ on channel *l*.

As shown in Figure 1, we first perform the GAP operation on the input feature $U_{m}$ to obtain the channel-level global feature $Z_{m}=\left[z_{m}^{1},z_{m}^{2},\cdots ,z_{m}^{C_{m}}\right]\in R^{1\times 1\times C_{m}}$. The operation expression on channel *l* ($l=1,\cdots ,C_{m}$) is shown as follows:

(1) $$\begin{array}{cc} z_{m}^{l} & =GAP(u_{m}^{l})\\ \; & =\frac{1}{H_{m}\times W_{m}}\sum_{i=1}^{H_{m}}\sum_{j=1}^{W_{m}}u_{m}^{l}\left(i,j\right). \end{array}$$

Next, we multiply the obtained channel-level global feature $Z_{m}$ with the original input feature $U_{m}$ as the feature enhancement operation. The enhanced feature is represented by $U_{m}^{\prime }=\left[u_{m}^{\prime 1},u_{m}^{\prime 2},\cdots ,u_{m}^{\prime C_{m}}\right]\in R^{H_{m}\times W_{m}\times C_{m}}$. The operation expression on channel *l* ($l=1,\cdots ,C_{m}$) is shown as follows:

(2) $$\begin{array}{c} u_{m}^{\prime l}=z_{m}^{l}\cdot u_{m}^{l}. \end{array}$$

The original feature can produce feature weights with a global receptive field after the GAP. If the feature weights and original feature are fused by channels, each channel of the original feature will learn global information, thus enriching the original feature and making the feature more expensive to realize. Finally, we put $U_{m}^{\prime }$ into the Conv Block to obtain the final enhanced feature ${\tilde{U}}_{m}\in R^{H_{m}\times W_{m}\times C_{m}}$, as shown in Figure 1.

### 2.2. Multi-Stage Features Fusion (MSF^2^)

After obtaining the enhanced feature ${\tilde{U}}_{m}\in R^{H_{m}\times W_{m}\times C_{m}}$ (m=2,3,4) of each stage, we perform the fusion operation on these features. First, we flatten each feature ${\tilde{U}}_{m}$ into a vector $v_{m}$ as follows:

(3) $$\begin{array}{c} v_{m}=F\left(AvgP\left({\tilde{U}}_{m}\right)\right)\in R^{C_{m}}. \end{array}$$

As shown in the above equation, we first use average pooling (AvgP) to map ${\tilde{U}}_{m}$ to the $1\times 1\times C_{m}$ dimension and perform a flattening operation (*F*) to map it to the $C_{m}$ dimension. Then, we fuse the flattened vectors of each stage, and its operations are shown as follows:

(4) $$\begin{array}{c} \tilde{v}=FC\left(\left[v_{2},v_{3},v_{4}\right]\right)\in R^{C^{\prime }}. \end{array}$$

As shown in the above equation, we first concatenate all $v_{m}$ into a new vector and use a fully connected layer (FC) to map it to the $C^{\prime }$ dimension.

Although this fusion method looks simple, it can keep the channel-wise global information learned after the FE Block at each stage well maintained. However, the information in the learned global features may be destroyed if other fusion methods are used.

### 2.3. Regularization

In this paper, we use a prototype conformity loss $L_{PC}$[26] proposed by Mustafa et al. as our regularization method. For a classification task with the number of classes *k*, given training images, let $f_{p}$ be the output feature of one image $x_{p}$ with class $y_{p}$. Therefore, the expression of $L_{PC}$ is shown as follows:

(5) $$\begin{array}{c} L_{PC}=\sum_{p}\{{∥f_{p}-w_{y_{p}}^{c}∥}_{2}-\frac{1}{k-1}\sum_{q\neq y_{p}}({∥f_{p}-w_{q}^{c}∥}_{2}+{∥w_{y_{p}}^{c}-w_{q}^{c}∥}_{2})\}, \end{array}$$

where $w_{y_{p}}^{c}$ is the class centroid corresponding to the true class $y_{p}$, and $w_{q}^{c}$ is the class centroids corresponding to other classes that are not class $y_{p}$. We can see from the above equation that $L_{PC}$ can increase the distance between different classes and reduce the distance between $f_{p}$ and the class center $w_{y_{p}}^{c}$; thus, the boundaries between different classes are more obvious.

It is easier for $L_{PC}$ to learn the differences among the features of different classes when the representation information of the features of each class is rich. Additionally, the output features of EMSF^2^Net contain information-rich global channel features. Naturally, we introduce $L_{PC}$ into our proposed network as the regularization method. Therefore, the total loss function $L_{all}$ used for training EMSF^2^Net is shown as follows:

(6) $$\begin{array}{c} L_{all}=L_{CE}+\sum_{k=1}^{4}L_{PC}^{k}, \end{array}$$

where the cross-entropy loss $L_{CE}$ is responsible for constraining the final classification outputs of EMSF^2^Net, and $L_{PC}$ aims to regularize the multi-stage features, and the enhanced multi-stage fusion feature in EMSF^2^Net. $\sum_{k=1}^{4}L_{PC}^{k}$ denotes the sum of all $L_{PC}$ in EMSF^2^Net. $L_{all}$ can increase the distances between samples with different classes and decrease the distances between samples with the same classes in the output space.

## 3. Dataset and Adversarial Attacks

In this section, we introduce the dataset and seven popular adversarial attack methods used in this paper to verify the adversarial robustness of our proposed method.

### 3.1. Dataset: CIFAR-10

We used the CIFAR-10 dataset [31], which has been widely used to verify adversarial defense methods, to compare our method with other state-of-the-art and ablation analysis methods. CIFAR-10 consists of 60,000 images with the size of 32 × 32 pixels; the training set contains 50,000 images, and the test set consists of 10,000 images. This dataset is divided into 10 classes: “airplane”, “automobile”, “bird”, “cat”, “deer”, “dog”, “frog”, “horse”, “ship”, and “truck”.

### 3.2. Attack Methods

Given a clean image x and its corresponding true label y, the model is represented as f, and the adversarial attack aims to find a perturbation η that human eyes cannot distinguish. This kind of perturbation should satisfy the following equation:

(7) $$\begin{array}{c} \underset{{∥\eta ∥}_{p}<\epsilon }{\mathrm{argmax}}L\left(f\left(x+\eta \right),y\right), \end{array}$$

where L represents the loss function; ${∥\cdot ∥}_{p}$ represents the $L_{p}$-norm with p∈{0,⋯,∞}, and ϵ is the perturbation or attack strength.

Currently, many adversarial attack methods for finding the perturbation have been proposed. In this paper, we used six popular adversarial attacks, which are shown in detail below, to evaluate the robustness of the proposed EMSF^2^Net. The adversarial attack toolbox used in the experiments is Torchattacks [32].

#### 3.2.1. Fast Gradient Sign Method

The fast gradient sign method (FGSM) [4] is a classic adversarial attack method. It generates the adversarial perturbation η based on the gradient of loss function of the clean image x. The generated adversarial example $x^{\prime }$ can be expressed as follows:

(8) $$\begin{array}{c} x^{\prime }=x+\epsilon \cdot \mathrm{sign}\left(\nabla_{x}L\left(f\left(x\right),y\right)\right), \end{array}$$

where ϵ represents the attack strength and the distance measure used for this attack is $L_{\infty }$.

#### 3.2.2. Projected Gradient Descent

Projected gradient descent (PGD) [5] is a kind of iterative adversarial attack method, which can be regarded as a kind of iteration FGSM. The expression of step k+1 is as follows:

(9) $$\begin{array}{cc} \; & x_{0}^{\prime }=x+U\left(-\epsilon ,\epsilon \right),\\ \; & x_{k+1}^{\prime }=P\left\{x_{k}^{\prime }+\alpha \cdot \mathrm{sign}\left[\nabla_{x_{k}^{\prime }}L\left(f(x_{k}^{\prime }),y\right)\right]\right\}, \end{array}$$

where U(·,·) is the uniform distribution, and α denotes the step size. The projection function P{·} guarantees that after each iteration, the generated adversarial example $x^{\prime }$ can always be in the ϵ-ball with x as the center, and ϵ is the radius. The distance measurements used for this attack are $L_{\infty }$ and $L_{2}$. Specifically, the PGD attack adopted the $L_{2}$-norm denoted as the PGD_$L_{2}$ in this paper.

#### 3.2.3. Momentum Iterative Fast Gradient Sign Method

The momentum iterative FGSM (MI-FGSM, MIM) [33] integrates momentum into the iteration process, which is unlike the traditional iteration-based FGSMs [5,34], and the expressions of step k+1 are shown as follows:

(10) $$\begin{array}{cc} \; & g_{0}=0,\;x_{0}^{\prime }=x,\\ \; & g_{k+1}=\mu \cdot g_{k}+\frac{\nabla_{x_{k}^{\prime }}L\left(f\left(x_{k}^{\prime }\right),y\right)}{{∥\nabla_{x_{k}^{\prime }}L\left(f\left(x_{k}^{\prime }\right),y\right)∥}_{1}},\\ \; & x_{k+1}^{\prime }=P\left\{x_{k}^{\prime }+\alpha \cdot \mathrm{sign}\left(g_{k+1}\right)\right\}, \end{array}$$

where μ is the decay factor for the gradient direction; α is the step size, and P{·} is the projection function that can project the generated adversarial example $x^{\prime }$ in the ϵ-ball. We used the $L_{\infty }$ distance measure for the MI-FGSM attack.

#### 3.2.4. Diverse Inputs Iterative Fast Gradient Sign Method

Inspired by data augmentation [35,36], the diverse inputs iterative FGSM (DI^2^-FGSM) [37] introduces the input diversity to improve the transferability of adversarial examples. Specifically, a random transformation function is designed to clean inputs and used in each iteration of generating adversarial examples. In this paper, we employ the momentum-based DI^2^-FGSM attack, and the expressions of step k+1 are shown as follows:

(11) $$\begin{array}{cc} \; & x_{0}^{\prime }=x+U\left(-\epsilon ,\epsilon \right),\\ \; & g_{k+1}=\mu \cdot g_{k}+\frac{\nabla_{x_{k}^{\prime }}L\left(f\left(T\left(x_{k}^{\prime };P\right)\right),y\right)}{{∥\nabla_{x_{k}^{\prime }}L\left(f\left(T\left(x_{k}^{\prime };P\right)\right),y\right)∥}_{1}},\\ \; & x_{k+1}^{\prime }=P\left\{x_{k}^{\prime }+\alpha \cdot \mathrm{sign}\left(g_{k+1}\right)\right\},\\ \; & T\left(x_{k}^{\prime };P\right)=\left\{\begin{array}{c} T(x_{k}^{\prime }),\mathrm{with}\;\mathrm{probability}\;P\\ x_{k}^{\prime },\mathrm{with}\;\mathrm{probability}\;1-P, \end{array}\right. \end{array}$$

Here, μ, α, and P{·} are defined the same as in Equation (10); T(·;·) is the random transformation function; and *P* is the transformation probability. We used the $L_{\infty }$-norm as the distance measurement of DI^2^-FGSM.

#### 3.2.5. Averaged Projected Gradient Descent

Inspired by expectation over transformation (EOT) [38], an averaged PGD (A-PGD, EOTPGD) [39] was proposed to obtain a more stable and effective adversarial attack than the vanilla PGD. It introduces the expectation into the PGD attack. The expressions on step k+1 of EOTPGD are shown as follows:

(12) $$\begin{array}{cc} \; & x_{0}^{\prime }=x+U\left(-\epsilon ,\epsilon \right),\\ \; & x_{k+1}^{\prime }=P\left\{x_{k}^{\prime }+\alpha \cdot \mathrm{sign}\left(E\left[\nabla_{x_{k}^{\prime }}L\left(f(x_{k}^{\prime }),y\right)\right]\right)\right\}, \end{array}$$

where $E\left[\cdot \right]$ and α denote the expectation and step size, respectively. We adopt the $L_{\infty }$-norm as the distance measure of the EOTPGD attack.

#### 3.2.6. Carlini and Wagner

Carlini and Wagner (CW) [40] is a novel optimization-based adversarial attack method. Specifically, a new variable w is introduced and optimized according to the following expressions to generate more deceptive adversarial examples:

(13) $$\begin{array}{cc} \; & w^{\prime }=\min_{w}{∥\frac{1}{2}\left(\tanh\left(w\right)+1\right)-x∥}_{2}^{2}+c\cdot G\left(\frac{1}{2}\left(\tanh\left(w\right)+1\right)\right),\\ \; & x^{\prime }=\frac{1}{2}\left(\tanh\left(w^{\prime }\right)+1\right),\\ \; & G\left(\cdot \right)=\max\left(f{\left(\cdot \right)}_{y}-\max_{i\neq y}f{\left(\cdot \right)}_{i},-\kappa \right), \end{array}$$

where *c* is a hyperparameter positively related to the strength of the generated adversarial examples, whereas κ is a confidence hyperparameter that can make the adversarial example $x^{\prime }$ become misclassified more easily. $f{\left(\cdot \right)}_{y}$ represents the output probability of the true label y, and $f{\left(\cdot \right)}_{i}$ represents the output probability of being misclassified. We used the $L_{2}$-norm distance measure for the CW attack.

## 4. Comparison Experiments

### 4.1. Comparison Methods

To fully verify the effectiveness and robustness of the proposed EMSF^2^Net, we chose three state-of-the-art methods.

MART [41]:

A novel loss function for adversarial defense is proposed in this method, which can pay more attention to the misclassified samples, thereby improving the adversarial robustness of the deep model.

RobNet [42]:

In RobNet, the authors focus on the network structure and introduce the neural architecture search (NAS) method into adversarial defense so that the robust network structures can be searched and designed.

BPFC [43]:

To simulate human visual processing, the authors impose a regularizer for consistent representation of the features learned from different quantized images in BPFC. This regularizer can significantly improve the adversarial robustness of the deep model.

### 4.2. Performance against Adversarial Attacks with $L_{\infty }$-Norm

In this subsection, we will demonstrate the robust accuracy results of the proposed EMSF^2^Net and the comparison methods under the adversarial attacks using the $L_{\infty }$-norm on the CIFAR-10 dataset. Specifically, we choose FGSM, PGD, MI-FGSM, DI^2^-FGSM, and EOTPGD with different attack strengths to show the superiority of the proposed approach. These $L_{\infty }$-norm attacks are set to white-box. The attack strengths of these attacks are set to 2/255, 4/255, 8/255, and 16/255.

First, we show the clean and robust accuracies against single-step FGSM attacks on the CIFAR-10 dataset. The results are presented in Table 2. As shown in Table 2, we can confirm that the proposed EMSF^2^Net outperforms the comparison methods under the FGSM attack and keeps a high classification accuracy in the scene with clean images.

Next, we show the robust classification accuracy under the iteration-based $L_{\infty }$-norm adversarial attacks with less complexity (iteration number = 10). For the convenience of distinction, we use PGD-10, MI-FGSM-10, DI^2^-FGSM-10, and EOTPGD-10 to denote these attacks with the iteration number of 10. The step size of these attacks is set to ϵ/10, where ϵ denotes the attack strength. For MI-FGSM-10 and DI^2^-FGSM-10, the parameter of the momentum factor is set to 0.5. For EOTPGD-10, the number for estimating the mean gradient is set to 5. The results are presented in Table 3. As shown in the table, we obtain that the proposed EMSF^2^Net still maintains the large advantages compared to the comparison methods under more difficult iteration-based adversarial attacks. In particular, the gaps between EMSF^2^Net and the other three comparison methods gradually increase as the attack strength ϵ gradually increases. This phenomenon further illustrates the robustness and effectiveness of the proposed approach.

Finally, we show the performance of the proposed EMSF^2^Net and the comparison methods under more complex iteration-based adversarial attacks (iteration number = 20) using the $L_{\infty }$-norm. We use PGD-20, MI-FGSM-20, DI^2^-FGSM-20, and EOTPGD-20 to denote these attacks with the iteration number of 20. Except for the iteration number, the other parameters in the attacks with more complexity are the same as those with less complexity. The robust accuracy results are presented in Table 4. From this table, we can conclude that the classification results of the comparison methods decrease significantly as the attack strength ϵ increases under more complex attacks. In contrast, the proposed EMSF^2^Net still maintains a high classification accuracy.

### 4.3. Performance against Adversarial Attacks with $L_{2}$-Norm

In Section 4.2, we present the classification accuracy results of the proposed EMSF^2^Net and three state-of-the-art comparison methods under white-box attacks with $L_{\infty }$-norm. These results reveal the robustness of EMSF^2^Net against $L_{\infty }$-norm attacks. In this subsection, we adopt another type of widely used adversarial attacks, the $L_{2}$-norm attacks, to further and more comprehensively verify the effectiveness of the proposed EMSF^2^Net. Specifically, we use PGD_$L_{2}$ attacks with different iteration numbers and CW attacks, where PGD_$L_{2}$-10, PGD_$L_{2}$-20, and PGD_$L_{2}$-40 represent PGD_$L_{2}$ attacks with the iteration numbers of 10, 20, and 40, respectively. Table 5 presents the robust accuracy results of the proposed EMSF^2^Net and the comparison methods under $L_{2}$-norm attacks with different attack strengths ϵ or different iteration numbers. The step size of the PGD_$L_{2}$ attacks is set to ϵ/10, and the parameter *c* for box-constraint and confidence κ in CW are set to 1.0 and 0, respectively. These $L_{2}$-norm attacks are set to white-box.

Table 5 shows that EMSF^2^Net can always maintain the highest accuracy under different $L_{2}$-norm attacks with different strengths and iterations compared to the comparison methods. In particular, the accuracy of the comparison methods drops rapidly, even lower than 1.0% in some cases, with the increase in ϵ under the PGD_$L_{2}$ attacks. In contrast, the proposed EMSF^2^Net can still maintain a relatively high adversarial robustness. The proposed EMSF^2^Net can also maintain the comparable performance under the notoriously difficult CW attack.

## 5. Ablation Analysis

In this section, we conducted a series of ablation experiments to further reveal the effectiveness and robustness of EMSF^2^Net.

We used two approaches for the ablation analysis. The first one is the baseline ResNet-50 shown in Table 1. We added three $L_{PC}$ at the outputs of STAGES 2–4 for a fair comparison. We also constructed a new architecture called MSF^2^Net (Multi-Stage Feature Fusion Network) to verify the effectiveness of the FE Block. Compared with EMSF^2^Net, MSF^2^Net removes the FE Block of each stage, and the remaining parts are the same as EMSF^2^Net. The total loss functions of the baseline and MSF^2^Net during the training process are the sum of $L_{CE}$ and $L_{PC}$.

First, in Section 5.1, we vividly show the performance of the baseline, MSF^2^Net, and EMSF^2^Net under the adversarial attacks with different parameter settings in the form of line graphs. Then, in Section 5.2, we present the classification accuracy of each class in the CIFAR-10 dataset for the three approaches against different attacks in the form of histograms to reveal which classes in the CIFAR-10 dataset are more likely to be misclassified using these methods. Furthermore, in Section 5.3, we use a powerful tool for interpretability, grad-cam, to visualize each stage (STAGES 1–4) of the three methods. We also reveal which features the three methods focus on under adversarial attacks. Thus, the reason for the adversarial robustness of the proposed EMSF^2^Net can be understood. Finally, we use another popular interpretability tool, t-SNE, to show the feature distributions of three approaches under adversarial attacks with different settings.

### 5.1. Performance on Three Methods

**Figure 2:**
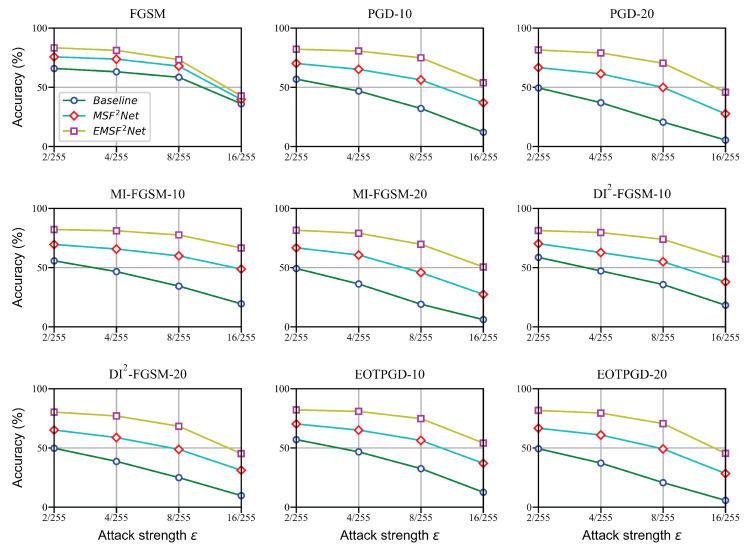
Robust classification accuracy of the baseline, MSF^2^Net, and EMSF^2^Net under adversarial attacks using the $L_{\infty }$-norm on the CIFAR-10 dataset.

**Figure 3:**
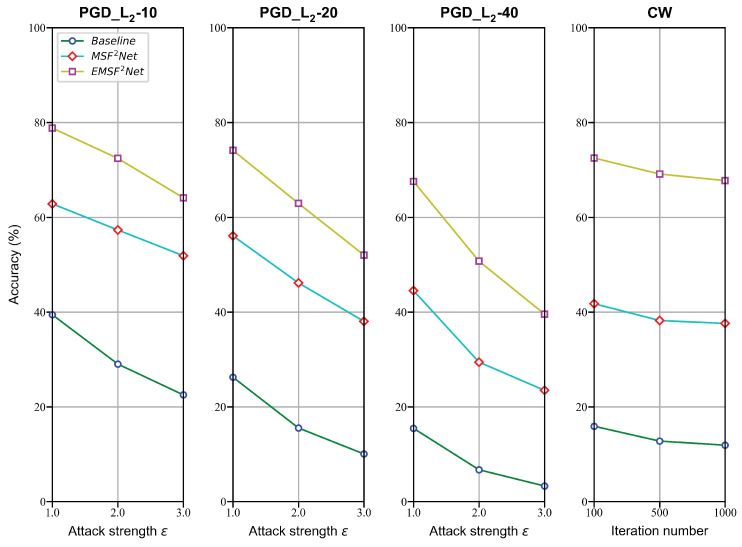
Robust classification accuracy of the baseline, MSF^2^Net, and EMSF^2^Net under adversarial attacks using the $L_{2}$-norm on the CIFAR-10 dataset.

In this subsection, we present the classification results of the baseline, MSF^2^Net, and EMSF^2^Net under the $L_{\infty }$-norm and $L_{2}$-norm attacks with the white-box setting on the CIFAR-10 dataset. First, the performance under the $L_{\infty }$-norm attacks is given and shown in Figure 2. The attack strengths ϵ of these attacks are set to 2/255, 4/255, 8/255, and 16/255, respectively. Other parameters are set the same as the parameters explained in Section 4.2. Next, we show the robust accuracy of these three methods under the $L_{2}$-norm white-box attacks in Figure 3. The attack strengths ϵ of PGD_$L_{2}$ attacks are set to 1.0, 2.0, and 3.0, whereas the iteration numbers of CW are set to 100, 500, and 1000, respectively. Other parameters are set the same as the parameters explained in Section 4.3.

As shown in Figure 2, although the gaps between the three approaches are not obvious under the FGSM attack, the advantages of the proposed EMSF^2^Net gradually emerge under the iteration-based attacks. Moreover, the proposed EMSF^2^Net still outperforms the baseline and MSF^2^Net under the $L_{2}$-norm white-box attacks. Particularly, the robust accuracy of the baseline and MSF^2^Net are below 50% under the CW attack, whereas the accuracy of the proposed EMSF^2^Net is consistently above 60%. Thus, the effectiveness of the FE Block is also clearly verified from Figure 2 and Figure 3.

### 5.2. Performance on Each Class of CIFAR-10

**Figure 4:**
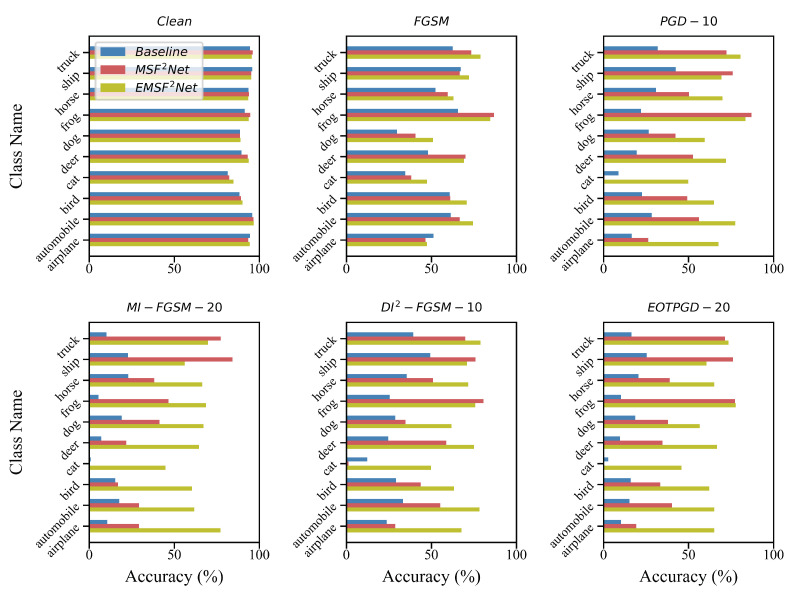
Clean and robust accuracies under $L_{\infty }$-norm attacks of the three methods on each class of the CIFAR-10 dataset. The X-axis and Y-axis of each sub-figure represent the robust accuracy and class name of CIFAR-10, respectively.

**Figure 5:**
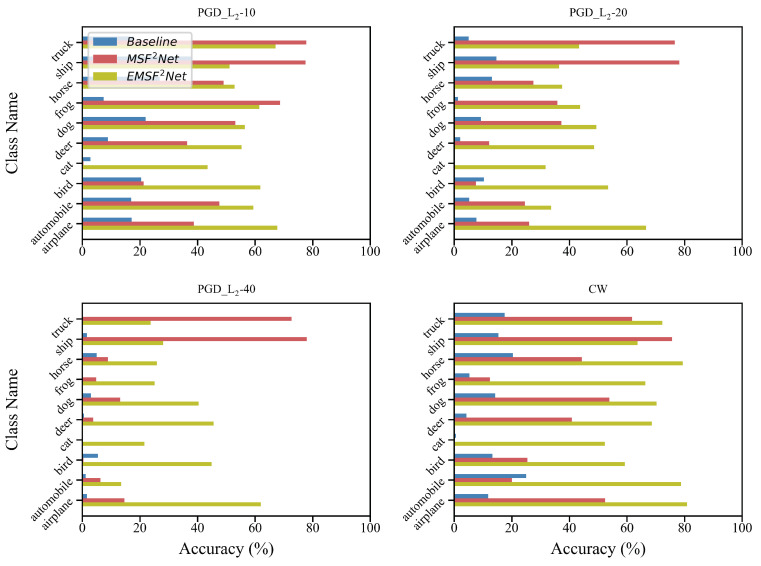
Robust accuracy under $L_{2}$-norm attacks of the three methods on each class of the CIFAR-10 dataset. The X-axis and Y-axis of each sub-figure represent the robust accuracy and class name of CIFAR-10, respectively.

To further investigate the impacts of white-box adversarial attacks, we output the accuracy of each class of the baseline, MSF^2^Net, and EMSF^2^Net, and the results are shown in Figure 4 and Figure 5. Figure 4 shows the clean accuracy of each class and the robust accuracy of each class under the $L_{\infty }$-norm white-box attacks, while Figure 5 shows the robust accuracy under the white-box attacks with the $L_{2}$-norm. In Figure 4, we use FGSM, PGD-10, MI-FGSM-20, DI^2^-FGSM-10, and EOTPGD-20 with the same attack strength ϵ=0.04. For PGD-10, the step size is set to 0.004. For MI-FGSM-20 and DI^2^-FGSM-10, their step size and momentum factor are set to 0.004 and 0.5, respectively. For EOTPGD-20, its step size and number for estimating the mean gradient are set to 0.004 and 5. In Figure 5, we use the PGD_$L_{2}$ attacks (PGD_$L_{2}$-10, PGD_$L_{2}$-20, and PGD_$L_{2}$-40) and CW attack. For the PGD_$L_{2}$ attacks, their attack strength and step size are set to 4.0 and 0.4, respectively. For CW, its box-constraint parameter *c*, confidence κ, and iteration number are set to 1.0, 0, and 400, respectively.

The upper left part of Figure 4 is the clean accuracy of each class. We can see that when there is no attack, the accuracy of each class of the three methods almost has no difference. However, after the adversarial attacks, the accuracy gaps between the three methods appear. As shown in Figure 4 and Figure 5, after the $L_{\infty }$- and $L_{2}$-norm attacks, EMSF^2^Net can always keep a comparable, or an even better, performance compared with the baseline and MSF^2^Net. In particular, the accuracy of baseline and MSF^2^Net on class “cat” is extremely low, but EMSF^2^Net still maintains high accuracy. Regarding the reason for this, we consider that the structural information contained in the images with class “cat” is more complicated than that contained in the images with other classes, and as mentioned earlier, the features after the FE Block in EMSF^2^Net will have a strong ability to express information. Therefore, they can better represent the information in the images with the class “cat”, while the features in the baseline and MSF^2^Net may not be able to represent this rich information well. So after regularization, the adversarial robustness of class “cat” will be weak.

### 5.3. Grad-Cam Visualization

In this subsection, to understand which features the three approaches pay attention to when facing adversarial attacks, we use grad-cam to visualize the output features of STAGES 1–4 in these three methods. In this way, the recognition mechanism of the three methods under adversarial attacks can be revealed. It is also possible to know why the proposed EMSF^2^Net can keep high robustness.

**Figure 6:**
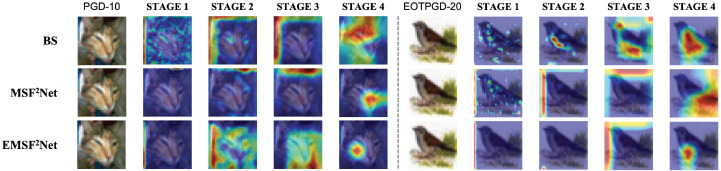
Grad-cam visualization results of the baseline, MSF^2^Net, and EMSF^2^Net under adversarial attacks with $L_{\infty }$-norm.

**Figure 7:**
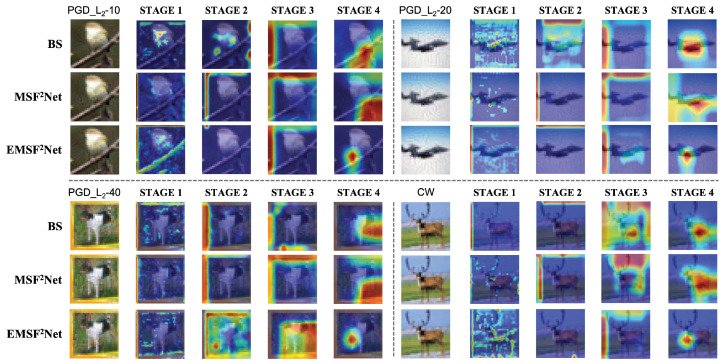
Grad-cam visualization results of the baseline, MSF^2^Net, and EMSF^2^Net under adversarial attacks with $L_{2}$-norm.

Figure 6 and Figure 7 show the visualization results under the white-box $L_{\infty }$-norm and white-box $L_{2}$-norm attacks, respectively. The “BS” in Figure 6 and Figure 7 denotes the baseline method. For the $L_{\infty }$-norm attacks, we use PGD-10 and EOTPGD-20, and their attack strength ϵ and step size are set to 0.02 and 0.002, respectively. Additionally, the parameter for estimating the mean gradient in EOTPGD-20 is set to 5. For the $L_{2}$-norm attacks, we adopt the PGD_$L_{2}$ attacks with different iterations (PGD_$L_{2}$-10, PGD_$L_{2}$-20, and PGD_$L_{2}$-40) and the CW attack, which is known for its difficulty. For the PGD_$L_{2}$ attacks, their attack strength and step size are set to 2.0 and 0.2, respectively. For CW, its box-constraint, confidence, and iteration parameters are set to 1.0, 0, and 500, respectively.

From Figure 6 and Figure 7, we can conclude that although the attention regions of STAGES 1–3 of the three approaches are confusing, the three methods begin to differ in the attention regions of STAGE 4. Specifically, the attention regions of the baseline and MSF^2^Net at STAGE 4 are either not the target class or are relatively large. However, the proposed EMSF^2^Net can always focus on the most important features of the target. We believe that for a non-denoising network, when the input is the adversarial image, it is easier to misclassify if the attention regions of the network are larger. This is because the texture features in the adversarial image have been contaminated, and if more regions are focused on, more erroneous features will be extracted. In contrast, if a network can always focus on and extract the most core features in the adversarial image, the classification accuracy can be improved since the core features contain relatively fewer adversarial noises.

Moreover, regarding which features in CNN are more important, as can be seen from Figure 6 and Figure 7, the areas of interest of STAGE 1–3 (shallow layers) are not the target areas. However, the outputs of STAGE 4 (deep layers) may affect the final classification results. Therefore, we can conclude that the deep layers are more important and more suitable for regularization.

### 5.4. t-SNE Visualization

**Figure 8:**
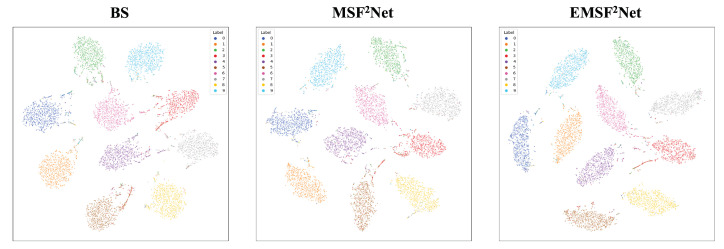
t-SNE visualization results of the baseline, MSF^2^Net, and EMSF^2^Net without any attacks on the CIFAR-10 dataset.

**Figure 9:**
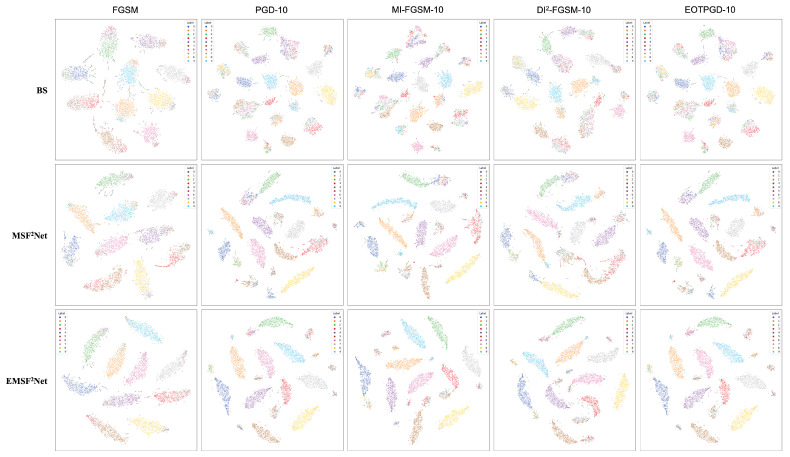
t-SNE visualization results of the baseline, MSF^2^Net, and EMSF^2^Net under the $L_{\infty }$-norm attacks with less complexity on the CIFAR-10 dataset.

**Figure 10:**
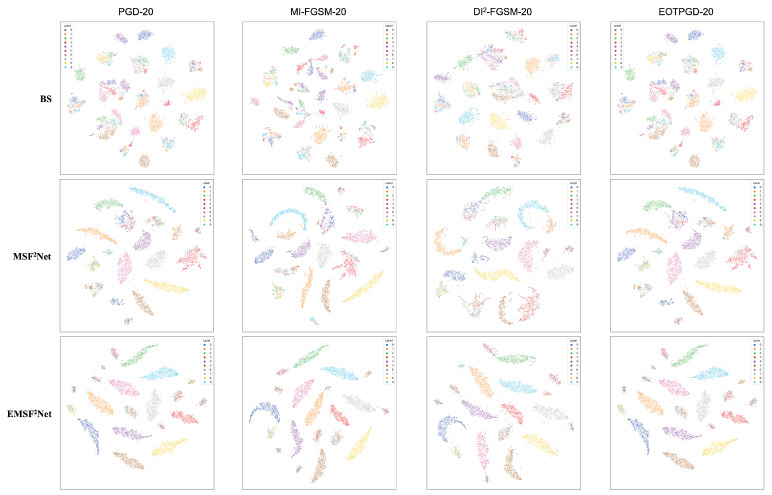
t-SNE visualization results of the baseline, MSF^2^Net, and EMSF^2^Net under the $L_{\infty }$-norm attacks with more complexity on the CIFAR-10 dataset.

**Figure 11:**
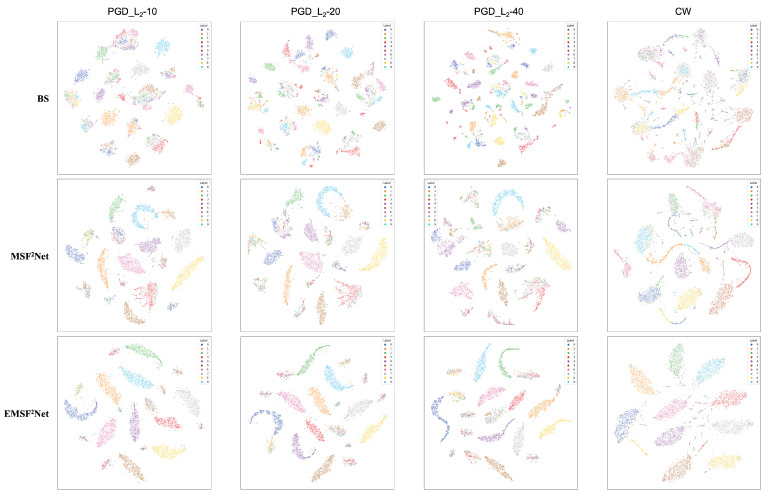
t-SNE visualization results of the baseline, MSF^2^Net, and EMSF^2^Net under the $L_{2}$-norm attacks on the CIFAR-10 dataset.

In this subsection, we use t-SNE to visualize the output features of the last layer in these three approaches to view the feature distributions of the baseline, MSF^2^Net, and EMSF^2^Net. Figure 8, Figure 9, Figure 10 and Figure 11 show the visualization results under no attacks, less complex $L_{\infty }$-norm attacks, more complex $L_{\infty }$-norm attacks, and $L_{2}$-norm attacks, respectively. In these figures, “BS” denotes the baseline method, and the adversarial attacks used here are white-box settings. The serial numbers 1–10 in these figures represent “airplane”, “automobile”, “bird”, “cat”, “deer”, “dog”, “frog”, “horse”, “ship”, and “truck” on the CIFAR-10 dataset, respectively.

In Figure 9, we adopt FGSM, PGD-10, MI-FGSM-10, DI^2^-FGSM-10, and EOTPGD-10 with the same attack strength ϵ=4/255 as $L_{\infty }$-norm attacks with less complexity. The step size, momentum factor, and number for estimating the mean gradient are set to ϵ/10, 0.5, and 5, respectively. For the more complex $L_{\infty }$-norm attacks in Figure 10, we use PGD-20, MI-FGSM-20, DI^2^-FGSM-20, and EOTPGD-20. The parameters, except the iteration number, are the same as the parameters used in Figure 9. Finally, we adopt the PGD_$L_{2}$ attacks with different iterations (PGD_$L_{2}$-10, PGD_$L_{2}$-20, and PGD_$L_{2}$-40) and the CW attack as the $L_{2}$-norm attacks in Figure 11. For the PGD_$L_{2}$ attacks, their attack strength ϵ and step size are set to 1.0 and 0.1, respectively. For CW, its box-constraint, confidence, and iteration parameters are set to 1.0, 0, and 50, respectively.

From Figure 8, we can conclude that the boundaries between each class of the CIFAR-10 dataset of the three methods are relatively obvious, indicating that these three methods can classify CIFAR-10 well without any attacks. However, the gaps between the three methods begin to appear under various adversarial attacks. From Figure 9, Figure 10 and Figure 11, we can find that under adversarial attacks, the classification results of the baseline are very chaotic, and the boundaries between each class of CIFAR-10 are very blurred; however, the performance of MSF^2^Net is slightly better. In contrast, the proposed EMSF^2^Net can always keep clear classification boundaries in most cases, which fully demonstrates the robustness and effectiveness of our method.

## 6. Conclusions

In this paper, we explored the adversarial defense based on regularization. We observe that the existing regularization-based adversarial defense methods do not discuss in detail what type of features are more suitable for regularization to further improve the adversarial robustness of CNNs. Therefore, we propose a new CNN architecture called EMSF^2^Net, consisting of three core operations: MSFE, MSF, and regularization. The proposed EMSF^2^Net shows that the robustness of CNN will be significantly improved if the enhanced multi-stage fusion feature is regularized. Extensive comparison experiments and ablation studies of white-box adversarial attacks with different settings demonstrate the effectiveness and robustness of our proposed method since the visual information processing mechanisms of different CNN-based structures are similar. Specifically, we believe that the CNN-based structures use operations such as convolution to extract the correlations between local data to effectively learn the representation information of each specific class. Thus, we have reason to believe that the proposed approach also performs well in other CNN-based structures. Regarding the performance of the proposed method on other structures, we would like to show it in future works. 

## Figures and Tables

**Table 1:** Architecture details of the baseline in our method. Among them, $L_{PC}$ represents the loss of the regularization method proposed by Mustafa et al. [26], and $L_{CE}$ represents the common cross-entropy loss.

*Layer*	ResNet50
STAGE 0	Conv(64,3×3),BN,ReLU
STAGE 1	$\left[\begin{array}{c} \mathrm{Conv}(64,1\times 1),\mathrm{BN},\mathrm{ReLU}\\ \mathrm{Conv}(64,3\times 3),\mathrm{BN},\mathrm{ReLU}\\ \mathrm{Conv}(256,1\times 1),\mathrm{BN}\\ \mathrm{shortcut},\mathrm{ReLU} \end{array}\right]$ ×3
STAGE 2	$\left[\begin{array}{c} \mathrm{Conv}(128,1\times 1),\mathrm{BN},\mathrm{ReLU}\\ \mathrm{Conv}(128,3\times 3),\mathrm{BN},\mathrm{ReLU}\\ \mathrm{Conv}(512,1\times 1),\mathrm{BN}\\ \mathrm{shortcut},\mathrm{ReLU} \end{array}\right]$ ×4 Maxpooling $\to L_{PC}$
STAGE 3	$\left[\begin{array}{c} \mathrm{Conv}(256,1\times 1),\mathrm{BN},\mathrm{ReLU}\\ \mathrm{Conv}(256,3\times 3),\mathrm{BN},\mathrm{ReLU}\\ \mathrm{Conv}(1024,1\times 1),\mathrm{BN}\\ \mathrm{shortcut},\mathrm{ReLU} \end{array}\right]$ ×6 Maxpooling $\to L_{PC}$
STAGE 4	$\left[\begin{array}{c} \mathrm{Conv}(512,1\times 1),\mathrm{BN},\mathrm{ReLU}\\ \mathrm{Conv}(512,3\times 3),\mathrm{BN},\mathrm{ReLU}\\ \mathrm{Conv}(2048,1\times 1),\mathrm{BN}\\ \mathrm{shortcut},\mathrm{ReLU} \end{array}\right]$ ×3
5	Averagepooling $\to L_{PC}$
6	FC(4096) $\to L_{PC}$
7	FC(10) $\to L_{CE}$

**Table 2:** Clean and robust accuracies under the FGSM attack on CIFAR-10 dataset. The lightgray row represents the results of the proposed method, and the bold results represent the best results.

		FGSM			
Method	No Attack	$\epsilon =\frac{2}{255}$	$\epsilon =\frac{4}{255}$	$\epsilon =\frac{8}{255}$	$\epsilon =\frac{16}{255}$
MART	83.6%	78.4%	72.9%	61.6%	42.6%
RobNet	82.7%	76.9%	70.6%	58.4%	38.0%
BPFC	82.4%	73.7%	64.6%	50.1%	33.7%
EMSF^2^Net (Ours)	**92.7%**	**83.3%**	**81.1%**	**73.3%**	**42.7%**

**Table 3:** Robust accuracy against $L_{\infty }$-norm attacks with less complexity on CIFAR-10 dataset. The lightgray row represents the results of the proposed method, and the bold results represent the best results.

	Attack Strength							
	$\epsilon =\frac{2}{255}$	$\epsilon =\frac{4}{255}$	$\epsilon =\frac{8}{255}$	$\epsilon =\frac{16}{255}$	$\epsilon =\frac{2}{255}$	$\epsilon =\frac{4}{255}$	$\epsilon =\frac{8}{255}$	$\epsilon =\frac{16}{255}$
	**PGD-10**				**MI-FGSM-10**			
MART	79.7%	75.6%	65.5%	43.3%	78.3%	72.4%	59.1%	32.9%
RobNet	78.3%	73.4%	62.1%	38.6%	76.8%	70.1%	55.7%	27.8%
BPFC	75.7%	68.2%	52.0%	26.9%	73.4%	63.2%	44.5%	20.5%
EMSF^2^Net (Ours)	**82.1%**	**80.6%**	**74.8%**	**53.8%**	**82.1%**	**81.0%**	**77.5%**	**66.5%**
1c	**DI^2^-FGSM-10**				**EOTPGD-10**			
MART	79.9%	76.1%	66.6%	45.8%	79.7%	75.6%	65.4%	43.5%
RobNet	78.6%	74.0%	63.3%	40.9%	78.3%	73.3%	62.1%	38.4%
BPFC	76.1%	69.4%	53.6%	29.1%	75.7%	68.3%	52.0%	26.9%
EMSF^2^Net (Ours)	**81.2%**	**79.5%**	**73.8%**	**57.2%**	**82.1%**	**80.7%**	**74.7%**	**54.1%**

**Table 4:** Robust accuracy against $L_{\infty }$-norm attacks with more complexity on the CIFAR-10 dataset. The lightgray row represents the results of the proposed method, and the bold results represent the best results.

	Attack Strength							
	$\epsilon =\frac{2}{255}$	$\epsilon =\frac{4}{255}$	$\epsilon =\frac{8}{255}$	$\epsilon =\frac{16}{255}$	$\epsilon =\frac{2}{255}$	$\epsilon =\frac{4}{255}$	$\epsilon =\frac{8}{255}$	$\epsilon =\frac{16}{255}$
	**PGD-20**				**MI-FGSM-20**			
MART	78.3%	72.2%	57.7%	27.7%	78.3%	72.0%	57.3%	26.6%
RobNet	76.7%	69.7%	53.9%	22.5%	76.7%	69.6%	53.5%	21.8%
BPFC	73.3%	61.9%	39.9%	13.0%	73.2%	61.8%	39.7%	12.9%
EMSF^2^Net (Ours)	**81.4%**	**79.0%**	**70.2%**	**45.9%**	**81.5%**	**79.0%**	**69.6%**	**50.5%**
1c	**DI^2^-FGSM-20**				**EOTPGD-20**			
MART	78.6%	73.0%	59.1%	30.1%	78.3%	72.2%	57.9%	27.6%
RobNet	77.0%	70.5%	55.5%	24.5%	76.7%	69.7%	53.7%	22.5%
BPFC	73.8%	63.5%	41.9%	14.5%	73.3%	62.0%	39.8%	13.1%
EMSF^2^Net (Ours)	**80.2%**	**76.9%**	**68.2%**	**45.1%**	**81.5%**	**79.4%**	**70.5%**	**45.4%**

**Table 5:** Robust accuracy against adversarial attacks using the $L_{2}$-norm on the CIFAR-10 dataset. The lightgray row represents the results of the proposed method, and the bold results represent the best results.

	Attack Strength					
	ϵ=1.0	ϵ=2.0	ϵ=3.0	ϵ=1.0	ϵ=2.0	ϵ=3.0
	**PGD_$L_{2}$-10**			**PGD_$L_{2}$-20**		
MART	46.2%	17.2%	4.7%	37.5%	5.5%	0.4%
RobNet	42.8%	14.4%	3.9%	34.5%	4.9%	0.5%
BPFC	47.1%	23.0%	11.3%	41.7%	12.9%	3.3%
EMSF^2^Net (Ours)	**78.8%**	**72.4%**	**64.1%**	**74.1%**	**62.9%**	**52.0%**
	**Attack strength**			**Iteration number** (steps)		
	ϵ=1.0	ϵ=2.0	ϵ=3.0	100	500	1000
	**PGD_$L_{2}$-40**			**CW**		
MART	34.1%	3.1%	0.1%	22.9%	21.0%	20.9%
RobNet	31.5%	3.1%	0.1%	12.9%	10.8%	10.8%
BPFC	39.4%	8.7%	1.4%	59.4%	59.4%	59.4%
EMSF^2^Net (Ours)	**67.6%**	**50.7%**	**39.6%**	**72.5%**	**69.1%**	**67.7%**

## Data Availability

A publicly available dataset was used in this work.
